# Intravenous administration of mesenchymal stem cells exerts therapeutic effects on parkinsonian model of rats: Focusing on neuroprotective effects of stromal cell-derived factor-1α

**DOI:** 10.1186/1471-2202-11-52

**Published:** 2010-04-26

**Authors:** Feifei Wang, Takao Yasuhara, Tetsuro Shingo, Masahiro Kameda, Naoki Tajiri, Wen Ji Yuan, Akihiko Kondo, Tomohito Kadota, Tanefumi Baba, Judith Thomas Tayra, Yoichiro Kikuchi, Yasuyuki Miyoshi, Isao Date

**Affiliations:** 1 Department of Neurological Surgery, Okayama University Graduate School of Medicine, Dentistry and Pharmaceutical Sciences, Okayama, Japan

## Abstract

**Background:**

Mesenchymal stem cells (MSCs) are pluripotent stem cells derived from bone marrow with secretory functions of various neurotrophic factors. Stromal cell-derived factor-1α (SDF-1α) is also reported as one of chemokines released from MSCs. In this research, the therapeutic effects of MSCs through SDF-1α were explored. 6-hydroxydopamine (6-OHDA, 20 μg) was injected into the right striatum of female SD rats with subsequent administration of GFP-labeled MSCs, fibroblasts, (i.v., 1 × 10^7 ^cells, respectively) or PBS at 2 hours after 6-OHDA injection. All rats were evaluated behaviorally with cylinder test and amphetamine-induced rotation test for 1 month with consequent euthanasia for immunohistochemical evaluations. Additionally, to explore the underlying mechanisms, neuroprotective effects of SDF-1α were explored using 6-OHDA-exposed PC12 cells by using dopamine (DA) assay and TdT-mediated dUTP-biotin nick-end labeling (TUNEL) staining.

**Results:**

Rats receiving MSC transplantation significantly ameliorated behaviorally both in cylinder test and amphetamine-induced rotation test compared with the control groups. Correspondingly, rats with MSCs displayed significant preservation in the density of tyrosine hydroxylase (TH)-positive fibers in the striatum and the number of TH-positive neurons in the substantia nigra pars compacta (SNc) compared to that of control rats. In the *in vitro *study, SDF-1α treatment increased DA release and suppressed cell death induced by 6-OHDA administration compared with the control groups.

**Conclusions:**

Consequently, MSC transplantation might exert neuroprotection on 6-OHDA-exposed dopaminergic neurons at least partly through anti-apoptotic effects of SDF-1α. The results demonstrate the potentials of intravenous MSC administration for clinical applications, although further explorations are required.

## Background

Parkinson's disease (PD) is a common neurological disorder characterized by degeneration of nigrostriatal dopaminergic neurons [1]. The neuronal loss leads to deficiency of DA in the striatum, which is responsible for characteristic motor symptoms such as akinesia, rigidity and tremor [2,3]. The medication using L-DOPA and surgical treatment such as deep brain stimulation are established as effective therapies, although those treatments might not repair the dopaminergic pathway or prevent its degeneration [4-6].

Cell therapy was developed as a hopeful therapeutic tool for PD. Neural stem cells [7,8], neural precursor cells [9], fetal cells [10,11] and embryonic stem cells [12] have been studied for treatment on PD model of rats. However, there are ethical problems about the usage of embryonic and fetal tissues. These cells are limited in availability and relatively difficult to be prepared. Adult mesenchymal stem cells (MSCs) have many advantages for cell therapy because of the easy availability and pluripotency without ethical problems [13,14].

Several cytokines are known to secrete from MSCs. SDF-1α is one of the chemotactic cytokines (chemokines) and the unique ligand for a CXC chemokine receptor (CXCR4) [15]. The chemokines induced by inflammation in the central nervous system (CNS) usually play a role in the local immune response. Meanwhile, recent studies showed that the central functions of chemokines are not restricted to neuroinflammation, as originally thought, but extend to novel functions [16-19]. SDF-1α was found to exert neuroprotective effects [20]. It suppressed cell loss of primary cortical cultures induced by H_2_O_2 _neurotoxicity with the modulation of neurotrophic factor-expression. Rats receiving intracerebral administration of SDF-1α reduced infarct volumes with functional amelioration through up-regulation of anti-apoptotic proteins [20].

Recent studies reported that intrastriatal transplantation of MSCs restored the function of nigrostriatal dopaminergic systems, leading to the early improvement of behavioral deterioration in PD model of rats [21-24]. However, there is no study that demonstrated therapeutic effects of intravenous MSC administration for PD model of rats. Furthermore, the mechanisms of functional recovery achieved by MSCs transplantation have not been revealed completely so far. In this study, we explored whether intravenous administration of MSCs exerted therapeutic effects on PD model of rats *in vivo*. Then, neuroprotective effects of SDF-1α secreted from transplanted MSCs were explored using 6-OHDA-exposed PC12 cells *in vitro*.

## Results

### *In vivo *study

#### Behavioral tests

**Figure 1:**
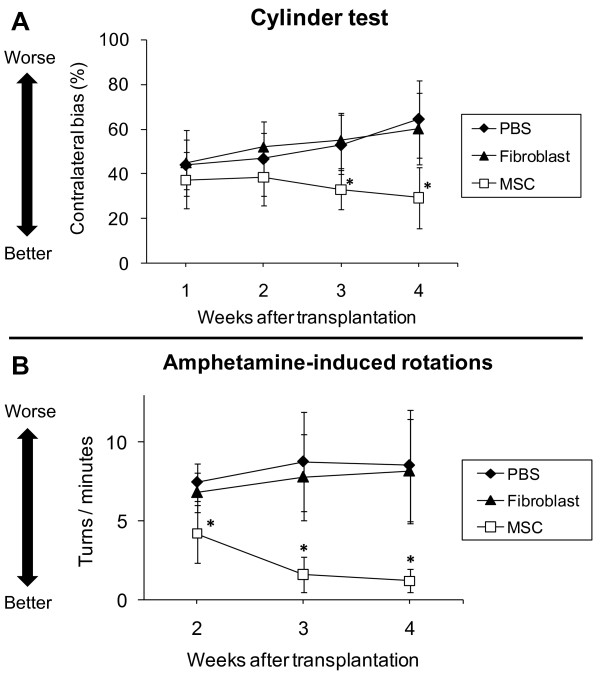
**Improvement in forelimb akinesia and amphetamine-induced rotations by MSC transplantation**. A: Cylinder test revealed the amelioration of forelimb akinesia in rats receiving MSC transplantation, compared with control groups at 3 and 4 weeks after transplantation. *p < 0.05 versus the control groups. B: The number of rotations was significantly less in rats receiving MSC transplantation, compared with that in control groups at 2, 3 and 4 weeks after transplantation. *p < 0.05 versus the control groups.

In cylinder test (Figure 1A), MSC group significantly ameliorated forelimb akinesia over time, compared to PBS and fibroblast group (PBS: 44.2 ± 11.3, 46.9 ± 16.6, 53.1 ± 13.3 and 64.7 ± 17.3%; fibroblast: 44.9 ± 14.8, 51.9 ± 6.3, 55.0 ± 12.4 and 60.2 ± 16.1%; MSC: 37.1 ± 12.5, 38.5 ± 12.6, 33.0 ± 8.9 and 29.3 ± 13.7% at 1, 2, 3 and 4 weeks after transplantation, respectively. One-way measures of ANOVA, F_2_, _16 _= 16.2, p < 0.0001 and post-hoc t-test of p's value < 0.01 vs. other control groups).

In amphetamine-induced rotation test (Figure 1B), MSC group significantly reduced the number of rotations over time, compared to PBS and fibroblast group (PBS: 7.5 ± 1.2, 8.8 ± 3.1 and 8.5 ± 3.5 turns/minute; fibroblast: 6.8 ± 1.2, 7.8 ± 2.7 and 8.2 ± 3.3 turns/minute; MSC: 4.2 ± 1.8, 1.6 ± 1.1 and 1.2 ± 0.7 turns/minute at 2, 3 and 4 weeks after transplantation, respectively. One-way measures of ANOVA, F_2_, _16 _= 15.8, p < 0.0001 and post-hoc t-test of p's value < 0.01 vs. other control groups). Thus, in the behavioral tests, MSC group ameliorated behaviorally, compared to fibroblast and PBS group with significant differences.

### TH Immunohistochemical staining in the striatum and SNc

**Figure 2:**
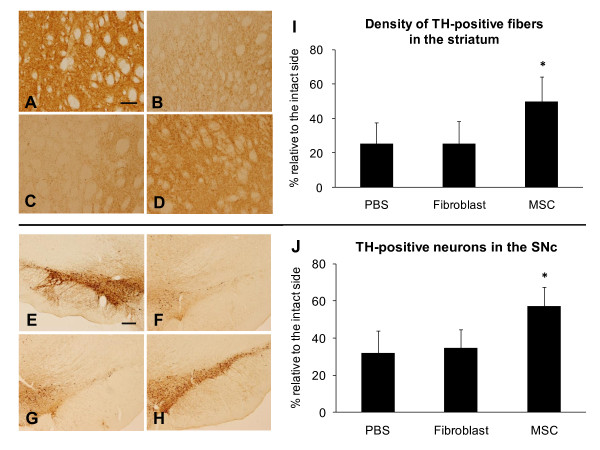
**Preservation of dopaminergic systems of 6-OHDA-lesioned rats by MSC transplantation**. A-H: TH-positive fibers in the striatum and neurons in the SNc of rats that received MSC transplantation were markedly preserved (D, H), compared with rats receiving PBS injection (B, F) and fibroblast transplantation (C, G). Normal TH-positive fibers and neurons were recognized in the intact striatum (A) and SNc (E). Scale bar: 100 μm in (A)-(D), 200 μm in (E)-(H). I: The density of TH-positive fibers in the striatum was analyzed with a computerized image analysis system. Rats receiving MSC transplantation showed a significant preservation in the density of TH-positive fibers in the striatum compared with the control rats. *p < 0.05 versus the control groups. J: The number of TH-positive neurons in the SNc was counted. The number of TH-positive neurons in the SNc was also significantly high, compared with the control rats. *p < 0.05 versus the control groups.

TH staining revealed the significant preservation of TH-positive fibers in the striatum (49.8 ± 14.5%, relative to the intact side, p values > 0.05, Figure 2A-D, I) and TH-positive cells in the SNc (57.3 ± 10.3%, relative to the intact side; p values > 0.01, Figure 2E-H, J) of MSC group, compared to those of fibroblast group (striatum: 25.2 ± 13.1%, SNc: 25.2 ± 13.1%) and PBS group (striatum: 25.3 ± 12.5%, SNc: 25.3 ± 12.5%).

#### Detection of transplanted MSCs

**Figure 3:**
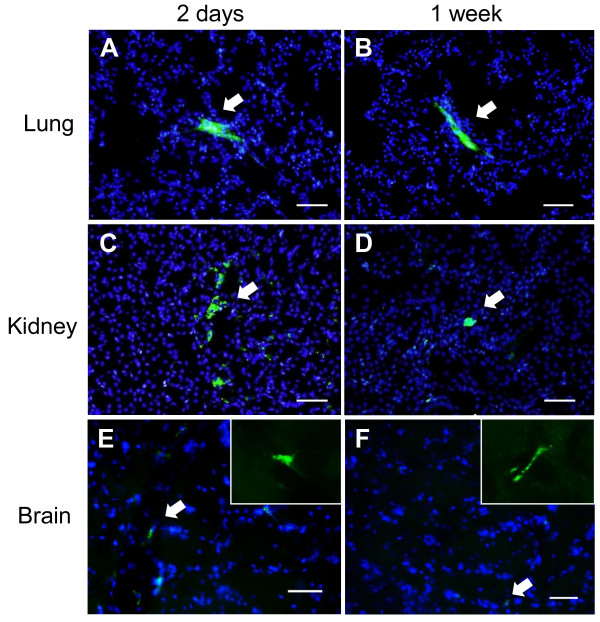
**Detection of transplanted MSCs**. Transplanted MSCs were observed in lung (A), kidney (C) and brain (E) at 2 days after transplantation. Some MSCs were also observed in lung (B), kidney (D) and brain (F) at 1 week after transplantation. The insets of panels E and F demonstrated the highly magnified images of GFP-positive MSCs. Green: GFP-positive MSCs, Blue: DAPI-positive nuclei. White arrows show transplanted GFP-MSCs. Scale bar: 50 μm.

At 2 days, 1 week and 4 weeks after cell transplantation, lungs, kidneys and brains were removed. We found that the majority of cells were localized to the pulmonary tissue and a few cells actually reached the brain and remained there at 2 days after transplantation (Figure 3A, C, E). Some cells were also observed in the pulmonary tissue and the brain at 1 week after transplantation (Figure 3B, D, F), there were significantly fewer than those seen at 2 days after transplantation. There were no detectable GFP-positive cells in the brain at 4 weeks after transplantation.

### *In vitro *study

#### Characterization of MSCs

**Figure 4:**
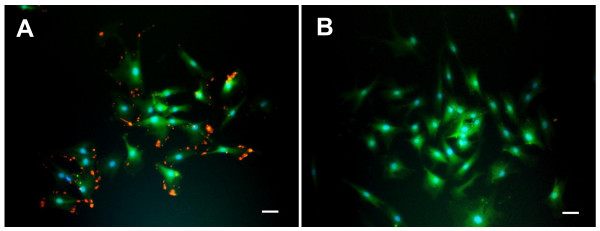
**Characterization of MSCs**. MSCs were positive for CD44 (CD44: red; GFP: green, A) and negative for CD45 (CD45: red; GFP: green, B). Scale bar: 50 μm.

We characterized MSCs by immunocytochemical investigations. MSCs were positive for matrix receptors, CD44 (93.2 ± 2.1%, Figure 4A) and negative for leukocyte common antigen, CD45 (3.8 ± 0.52%, Figure 4B).

#### Secretion of SDF-1α from MSCs

We used ELISA to determine whether SDF-1α was secreted from the MSCs. Over 6 and 24 hours period in culture, MSCs secreted 72.0 ± 16 pg/1 × 10^4 ^cells/6 hours and 183.5 ± 142 pg/1 × 10^4 ^cells/24 hours of SDF-1α.

#### CXCR4 staining

**Figure 5:**
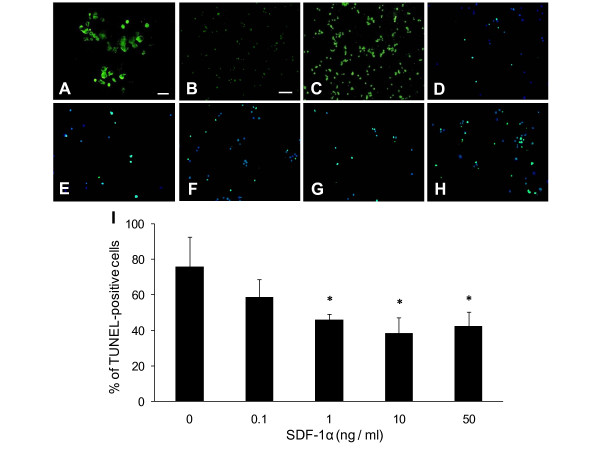
**Anti-apoptotic effects of SDF-1α on 6-OHDA-exposed PC12 cells**. A: CXCR4 was expressed in PBS-exposed PC12 cells. Scale bar: 20 μm. B, C: At 6 hours after 6-OHDA exposure, the expression of CXCR4 was remarkably up-regulated on 6-OHDA-exposed PC12 cells (C) compared with PBS-exposed PC12 cells (B). Scale bar: 100 μm. D-H: TUNEL/DAPI staining revealed that the number of apoptotic cells with green-colored, fragmented TUNEL-positive nuclei were reduced in SDF-1α treatment cells (D: 0.1 ng/ml, E: 1 ng/ml, F: 10 ng/ml, G: 50 ng/ml) compared with that of the control cells (H). Scale bar: 100 μm. I: SDF-1α treatment significantly reduced the number of TUNEL-positive 6-OHDA-exposed PC12 cells. *p < 0.05 versus the control with PBS.

CXCR4 staining revealed that normal PC12 cells expressed CXCR4 (Figure 5A). The expression of CXCR4 was also observed in 6-OHDA-exposed PC12 cells at 1, 6 and 12 hours after 6-OHDA exposure. At 6 hours after 6-OHDA exposure, the expression of CXCR4 was remarkably up-regulated on 6-OHDA-exposed PC12 cells compared with PBS-exposed PC12 cells (Figure 5B-C).

#### TUNEL staining

Nuclear fragmentation was observed in 6-OHDA-exposed PC12 cells at 12 hours after 6-OHDA exposure. The cell viability of PC12 cells was significantly increased by SDF-1α treatment (Figure 5D-H). Treatment with PBS showed many TUNEL-positive apoptotic cells (72.5 ± 8.2% relative to the whole cells). Treatment with 1, 10 and 50 ng/ml of SDF-1α significantly reduced apoptotic cell death (45.3 ± 2.6, 38.5 ± 8.8 and 42.6 ± 7.6%, respectively, p < 0.05, Figure 5I) compared to treatment with PBS. There were no significant differences among the different concentrations of SDF-1α, that is, 1, 10 and 50 ng/ml.

#### HPLC analysis

**Figure 6:**
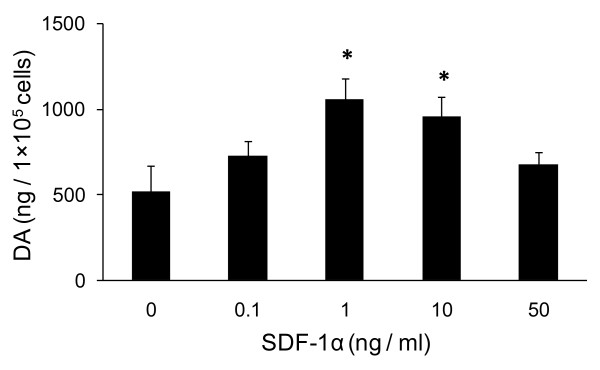
**Enhanced DA secretion from PC12 cells by SDF-1α**. PC12 cells treated with SDF-1α significantly increased DA secretion. *p < 0.05 versus the control with PBS.

The supernatant containing DA from 6-OHDA-exposed PC12 cells were analyzed with HPLC. DA release from 6-OHDA-exposed PC12 cells significantly increased by treatment with 1, 10 ng/ml of SDF-1α (1056 ± 126 ng/2 × 10^5 ^cells, 958 ± 114 ng/2 × 10^5 ^cells, respectively) compared to treatment with PBS (518 ± 150 ng/2 × 10^5 ^cells, Figure 6). There were no significant differences between the concentrations of 1 and 10 ng/ml of SDF-1α.

#### Neutralization assay for anti-apoptotic effect of SDF-1α

**Figure 7:**
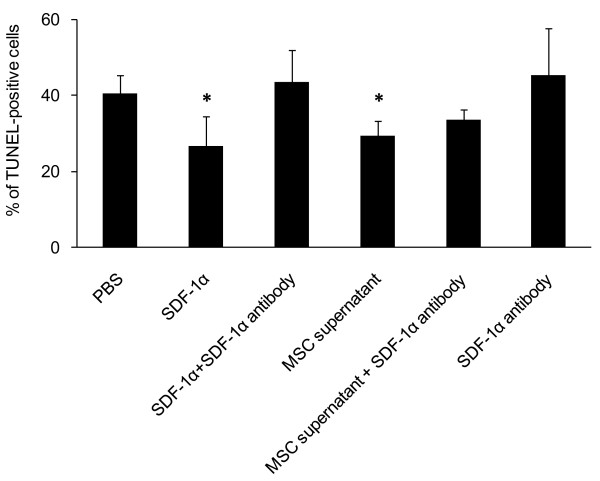
**Blockade of SDF-1α cancelled neuroprotective effects of SDF-1α on 6-OHDA-exposed PC12 cells**. SDF-1α antibody blocked the anti-apoptotic effect of SDF-1α and MSC supernatant exerted neuroprotective effects on 6-OHDA-exposed PC12 cells. *p < 0.05 versus all the control groups.

6-OHDA-exposed PC12 cells treated with SDF-1α or MSC supernatant showed significant suppression of apoptosis (26.6 ± 7.9 and 29.4 ± 3.7%, respectively) compared with PC12 cells treated with SDF-1α combined with anti-SDF-1α antibody, only anti-SDF-1α antibody, MSC supernatant combined with anti-SDF-1α antibody and PBS (43.4 ± 8.5, 45.3 ± 12.5, 33.5 ± 2.7 and 40.5 ± 4.7%, respectively, Figure 7). These results suggested that MSC supernatant exerted anti-apoptotic effects on 6-OHDA-exposed PC12 cells and SDF-1α antibody blocked the anti-apoptotic effect of SDF-1α.

## Discussion

We carried out intravenous administration of MSCs on PD model of rats and evaluated its therapeutic effects focusing on SDF-1α. Rats with MSC transplantation significantly ameliorated both in cylinder test and amphetamine-induced rotation test, compared with fibroblasts and PBS group. Correspondingly, rats with MSCs showed better preservation in the density of TH-positive fibers in the striatum and the number of TH-positive neurons in the SNc. To explore the underlying mechanisms, focusing on SDF-1α, we then proceeded to *in vitro *studies. In the *in vitro *studies, SDF-1α treatment suppressed apoptotic cell death of 6-OHDA-exposed PC12 cells with consequent increase of DA release from the cells, compared with the control. Furthermore, anti-SDF-1α antibody reduced the anti-apoptotic effects of SDF-1α.

### Anti-apoptotic effects of SDF-1α

In our present study, we demonstrated SDF-1α had anti-apoptotic effects against 6-OHDA-exposed PC12 cells *in vitro*. PC12 cells expressed CXCR4, an exclusive receptor of SDF-1α. CXCR4 is a member of G protein-coupled receptor (GPCR) family. Many studies have shown that SDF-1α/CXCR4 interaction causes activation of multiple signal transduction pathways, including phosphatidylinositol 3-kinase (PI3K)/Akt signaling pathway which provides enhanced survival signal and extracellular signal-regulated kinase (ERK 1/2) signaling pathway which provides enhanced cell proliferation [29]. A recent report also demonstrated that SDF-1α treatment reduced apoptotic cell death of endothelial progenitor cells under serum deprivation through PI3K/Akt pathway and decreased caspase-3-activity, an important apoptotic molecule [30].

In the CNS diseases, intracerebral administration of SDF-1α (1 μg/μl) exerted neuroprotection on stroke model of rats with reduced infarct volumes and improvement in neural plasticity. The report showed that SDF-1α disrupted the downstream of caspase-3 apoptotic signal in the ischemic penumbra of stroke model of rats with subsequent cortical neuronal protection [20]. In PD model of rats, caspase-3 activity is deeply involved in the apoptosis of dopaminergic neurons induced by 6-OHDA [31,32]. Therefore SDF-1α might enhance the survival of dopaminergic neurons through CXCR4, inducing down-regulation of caspase-3 and activation of PI3/Akt pathway.

### The activation of DA release by SDF-1α treatment

We showed that SDF-1α promoted DA release from 6-OHDA-exposed PC12 cells. A recent report demonstrated that CXCR4 was constitutively expressed on dopaminergic neurons in the SNc of normal rats and that SDF-1α could increase secretion of DA from dopaminergic neurons [33]. Thus, the results suggest that SDF-1α might activate nigrostriatal DA transmission. In our study, DA release might be increased by the functional preservation of 6-OHDA-exposed PC12 cells, as well as the enhanced survival of PC12 cells by SDF-1α, although the increasing effects on DA release might not depend on the dose of SDF-1α. Meanwhile, high dose of SDF-1α might have proapoptotic effects on the cells. Studies on neuroblastoma cells demonstrated that 10 nM of SDF-1α induced neuronal apoptosis after 24 hours-incubation by the activation of signaling pathways involving Src phosphorylation [34]. The discrepancy might lie on the differences of the used dosage, cells and experiment design, although we should know the fact that SDF-1α has neuroprotective effects with possible neuronal toxicity at high dose.

### Neuroprotective/neurotrophic effects of MSCs

Recently the therapeutic potentials of MSC transplantation have been studied in various pathological conditions of the CNS [35,36]. It have been demonstrated that intravenous administration of MSCs increased the expression of basic fibroblast growth factor (bFGF), suppressed apoptotic cell death, promoted endogenous cell proliferation and subsequently achieved functional recovery after stroke [37]. Additionally, MSCs produce neurotrophic factors, including vascular endothelial growth factor (VEGF), glial cell line-derived neurotrophic factor (GDNF) and brain-derived neurotrophic factor (BDNF), which are also well known as strong neuroprotectants [38]. The mechanisms of therapeutic effects of MSCs in the ischemic brain might include neuroprotective effects through the secreted neurotrophic factors, effective angiogenesis for amelioration in the microenvironment of ischemic penumbra and enhanced neurogenesis with possible neuronal differentiation of transplanted MSCs for synapse formation, although the number might be very low, especially with intravenous administration [39,40]. MSC transplantation might also exert neuroprotective effects on PD model of rats at least partly through the secreted trophic factors [41].

In our study, fluorescence microscopy revealed no detectable GFP-positive cells in the brain at 4 weeks after transplantation. This fact might be supposed from previous studies demonstrating that there were scant cells in the brain after intravenous transplantation of MSCs [39,40], although in our study, transplanted MSCs might die during the disease progression or lose green fluorescence before euthanasia. Nevertheless, MSC transplantation exerted strong therapeutic effects, which might be the proof that secreted trophic factors from MSC grafts might play a key role in the neuroprotective effects in our study. Additionally, MSCs were transplanted intravenously at 2 hours after 6-OHDA injection. The early transplantation of MSCs might be the reason of the strong therapeutic effects with the possibility to counteract 6-OHDA toxicity. As shown in the *in vitro *study, SDF-1α from MSCs probably contributed to the functional recovery as well as other trophic factors.

## Conclusions

The present data provided evidences that MSCs might exert neuroprotection for 6-OHDA-exposed dopaminergic neurons both *in vitro *and *in vivo *through possibly with anti-apoptotic mechanisms. The results suggest the potentials of intravenous MSC administration for PD. SDF-1α might be at least partly involved in the neuroprotective effects. Clinical application of MSC transplantation for PD patients might be considered, although further explorations are required.

## Methods

### *In vivo *study

#### Isolation, characterization and labeling of MSCs

We used adult female Sprague-Dawley rats (n = 6, 220-250 g, Charles River, Japan) according to the approved Guidelines of the Institutional Animal Care and Use Committee of Okayama University. Rats were euthanized by sodium pentobarbital (200 mg/kg) with subsequent removal of the femoral bones. After flushing of femoral bone marrow, total nucleated cells were cultured at 2 × 10^4 ^cells/cm^2 ^in DMEM (Gibco, Cergy Pontoise, France) supplemented with 10% fetal calf serum (Gibco, Cergy Pontoise, France), 1% (v/v) penicillin/streptomycin (Gibco, Cergy Pontoise, France). Fibroblasts were also obtained from rat skin and cultured in same medium. Cells were cultured at 37°C in a fully humidified atmosphere with 10% CO_2_. Medium was changed twice a week until confluence. The MSCs were isolated on the basis of their ability to adhere to the culture plates. After the second passage, expanded MSCs and fibroblast were used for infection with a fiber-mutant F/RGD adenovirus vector containing enhanced green fluorescent protein (EGFP) genes (AxCA-EGFP-F/RGD). Characterization of MSCs was assessed by immunocytochemical staining. MSCs were stained with antibodies against CD44, a representative marker of mesenchymal stem cells or CD45, a leukocyte common antigen [25,26]. All the stained cells were finally double stained with DAPI (4',6-diamino-2-phenylindole, Sigma-Aldrich, Tokyo, Japan).

#### PD model by intrastriatal injection of 6-hydroxydopamine

Adult female Sprague-Dawley rats weighing 220-250 g (n = 25, Charles River, kanagawa, Japan) were used for the experiments. All the rats were anesthetized by intraperitoneal injection of sodium pentobarbital (30 mg/kg) and placed on a stereotaxic apparatus (Narishige, Tokyo, Japan). The skull was exposed and a burr hole was drilled. A total of 20 μg of 6-OHDA (4 μl of 6-OHDA dissolved in saline containing 0.2 mg/ml ascorbic acid; Sigma-Aldrich, Tokyo, Japan) was administered in the right striatum with a Hamilton syringe (26 gauge, Hamilton, Massy, France) at a flow rate of 1 μl/min. Coordinates from the bregma were: AP = +1.0 mm, ML = +3.0 mm, DV = -5.0 mm. The syringe was left in place for 5 minutes after injection and then removed slowly to optimize toxin diffusion.

#### Intravenous administration of MSCs, fibroblasts or PBS

At 2 hours after 6-OHDA lesion, rats were anesthetized with 1.5% halothane with oxygen/nitrogen. GFP-labeled MSCs (n = 6), GFP-labeled fibroblasts (n = 6), (1 × 10^7 ^cells, respectively) or PBS (n = 7) were administered to PD model of rats by injection into the femoral vein.

#### Behavioral tests

All rats were evaluated behaviorally with cylinder test and amphetamine-induced rotation test for 1 month.

Forelimb akinesia was analyzed with cylinder test at 1, 2, 3 and 4 weeks after transplantation. Contacts made by each forepaw with the wall of a clear cylinder (diameter: 20 cm) were counted. The asymmetry score of forelimb use in wall exploration was calculated as a contralateral bias, where contralateral bias = [(the number of contacts with contralateral limb) - (the number of contacts with ipsilateral limb)]/[(the number of contacts with contralateral limb) + (the number of contacts with ipsilateral limb)] × 100 [27,28].

Amphetamine-induced rotational behavior was performed at 2, 3 and 4 weeks after transplantation. Rats were monitored for 90 minutes with a video camera after intraperitoneal injection of D-amphetamine (3.0 mg/kg, Dainippon Sumitomo Pharma, Osaka, Japan). Data are expressed as the number of complete body turns per minute.

#### Preparation of brain sections

At 4 weeks after transplantation, rats were euthanized and brains were removed. Coronal sections were cut at 40 μm-thickness with a freezing microtome (-20°C). Immunohistochemical investigations were performed with consecutive sections of the striatum and SNc.

#### TH Immunohistochemical staining in the striatum and SNc

TH staining was performed by free-floating methods. Sections were washed in PBS and incubated in 3% H_2_O_2 _in 70% methanol for 10 minutes to block the endogenous peroxidase activity. After washing in PBS, sections were incubated in rabbit anti-TH antibody solution (Chemicon, CA, 1:1000) with 10% normal goat serum for 18 hours at 4°C. After rinses in PBS, the sections were incubated for 30 minutes in biotin-conjugated donkey anti-rabbit IgG (Jackson Laboratories, CA, 1:1000) and subsequently avidin-peroxidase complex (Vector Laboratories, CA, 1:200) for 30 minutes. The sections were then treated with 3,4-diaminobenzidine (Sigma-Aldrich, Tokyo, Japan) and H_2_O_2_, mounted on albumin-coated slides, and sealed. Stained sections were analyzed with standard bright field microscopy (Olympus, Tokyo, Japan).

#### Detection of transplanted MSCs

At 2 days (n = 2), 1 week (n = 2) and 4 weeks (n = 2) after cell transplantation, rats were euthanized and brains, lungs and kidneys were removed in order to find where transplanted MSCs resided. Coronal sections were cut at 40 μm-thickness with a freezing microtome (-20°C). The sections were incubated with DAPI solution (Sigma-Aldrich, Tokyo, Japan) for 30 minutes. Finally, stained sections were analyzed with fluorescence microscopy (Keyence, Osaka, Japan).

### *In vitro *study

#### ELISA analysis for SDF-1α secretion

The level of SDF-1α secreted from MSCs was quantified using ELISA kits (R&D Systems, Minneapolis, MN). 1 × 10^4 ^MSCs were plated in 12-well culture plate (Thermo Fisher Scientific, Roskilde, Denmark). At 24 hours after plating for attachment, cells were incubated with Hanks' Balanced Salt Solutions (HBSS). Each 50 μl of the supernatant was obtained 6 and 24 hours later. This ELISA system can detect a minimum of 14 pg/ml of SDF-1α.

#### Culture of PC12 Cells

PC12 cells were obtained from Dainippon Sumitomo Pharma Biomedical (Osaka, Japan) and used for *in vitro *studies. Cells were plated in DMEM supplemented with 5% horse serum (Gibco, Cergy Pontoise, France), 5% newborn calf serum (Gibco, Cergy Pontoise, France), 1% (v/v) penicillin/streptomycin (Gibco, Cergy Pontoise, France) a density of 1 × 10^5 ^cells/well on poly-D-ornitin-coated glass slides in 24-well culture plate (Thermo Fisher Scientific, Roskilde, Denmark). Cells were cultured at 37°C in a fully humidified atmosphere with 5% CO_2_. At 24 hours after the initial plating, the medium was exchanged for fresh medium.

#### Addition of SDF-1α and 6-OHDA to PC12 cells

Human recombinant SDF-1α (Peprotech, NJ) was dissolved in PBS. PC12 cells were treated with each concentration of SDF-1α (0, 0.1, 1, 10, 50 ng/ml). At 30 minutes after SDF-1α treatment, cells were exposed to 50 μM 6-OHDA or PBS for 12 hours at 37°C. Then cells were fixed with 4% paraformaldehyde for immunocytochemical investigations. For HPLC analyses, at 12 hours after 6-OHDA exposure, cells were washed by HBSS twice. 500 μl of HBSS were put on each well for 1 hour. The supernatant was collected, filtered and stored at -80°C.

#### CXCR4 staining

To confirm the expression of CXCR4 in PC12 cells, we performed immunocytochemical investigations. At 24 hours after plating for attachment, cells were exposed to 50 μM of 6-OHDA or PBS for 1, 6 and 12 hours at 37°C. After exposure, cells were fixed with 4% paraformaldehyde and incubated in rabbit anti-CXCR4 antibody solution (Chemicon, MA, 1:1000) with 10% normal goat serum for 18 hours at 4°C. After rinses in PBS, the sections were incubated for 30 minutes in FITC-conjugated donkey anti-rabbit IgG (Jackson Laboratories, CA, 1:1000). Cells were mounted on the slides with GEL/MOUNT (Biomeda Corporation, CA) and analyzed with fluorescence microscopy (Olympus, Tokyo, Japan).

#### TUNEL staining

DNA fragmentation of apoptotic cells were identified by TUNEL method. The procedure was conducted according to the manufacturer's instruction (Roche, Basel, Switzerland). Briefly, PC12 cells were fixed with 4% paraformaldehyde for 30 minutes and washed in PBS 3 times. Then the cells were incubated with 3% H_2_O_2 _in 70% methanol for 10 minutes and washed with PBS twice. After that, the cells were incubated with 0.1% Triton X-100 in 0.1% sodium citrate for 2 minutes on ice. The slides were washed twice with PBS and the areas around samples were dried. TUNEL reaction mixture (50 μl) was added to each sample. Subsequently, slides were incubated for 60 minutes at 37°C in a humidified atmosphere in the dark. After washing with PBS, slides were mounted by GEL/MOUNT. Stained sections were analyzed with fluorescence microscopy (Olympus, Tokyo, Japan).

#### HPLC analysis

DA release from PC12 cells was analyzed by high performance liquid chromatography with electrochemical detection (HPLC-ECD, Shiseido Fine Chemicals, Tokyo, Japan). Aliquots (200 μl of HBSS) of the supernatant was injected into HPLC and interphased with an electrochemical detector with a glossy carbon flow-through detector cell. The analytes were separated on a C18, that is, 5 μm base deactivated reverse-phase column (Shiseido Fine Chemicals, Tokyo, Japan). The mobile phase with final pH of 2.5, consisted of 0.1 M NaH_2_PO_4_, 150 mg/L octyl sodium sulfate, 10 mg/L ethylenediamine tetraacetic acid (EDTA) disodium salt, and 5% acetonitrile.

#### Neutralization of anti-apoptotic effects by SDF-1α using anti-SDF-1α antibody

We explored whether the neutralization of SDF-1α might inhibit the anti-apoptotic effects of SDF-1α and supernatant of MSC culture might have anti-apoptotic effects on 6-OHDA-exposed PC12 cells. Human recombinant SDF-1α (Peprotech, NJ, 10 ng/ml) and rabbit anti-SDF-1α antibody (Peprotech, NJ, 0.5 μg/ml) were dissolved in PBS, respectively. Culture supernatant was prepared by harvesting MSCs (2 × 10^5 ^cells) in HBSS for 24 hours and diluted 33% to make the dose of SDF-1α appropriate for the results of previous reports. At 24 hours after plating, PC12 cells were treated with SDF-1α, SDF-1α combined with anti-SDF-1α antibody, only anti-SDF-1α antibody, MSC supernatant, MSC supernatant combined with anti-SDF-1α antibody and PBS. At 30 minutes after treatment, cells were exposed to 50 μM 6-OHDA or PBS for 12 hours at 37°C. After exposure, cells were washed 3 times in PBS and fixed for TUNEL assay.

### Statistical analysis

All values were expressed as mean ± standard deviation. Data were statistically analyzed by one-way measures of ANOVA. Statistical significance was present at p < 0.05.

## Authors' contributions

FW is involved in acquisition of all data and drafting/revising the manuscript. TS, TY, YM and ID designed the study, analyzed the data and revised the manuscript. NT and TK helped *in vivo *experiments including surgeries and animal care. WJY and AK helped *in vitro *experiments including immunocytochemical investigations. TB and YK helped immunohistochemical investigations. TJT and MK helped additional experiments in the revised manuscript. All authors read and approved the final manuscript.
